# Linking Digital Traits from Facial Expression, Voice, and Head Motion to Montgomery–Åsberg Depression Rating Scale Subscales

**DOI:** 10.1192/j.eurpsy.2024.576

**Published:** 2024-08-27

**Authors:** Z. Zhu, Y. Wu, J. Seidel, D. Roy, E. Salzmann

**Affiliations:** ^1^Boehringer Ingelheim Pharmaceuticals, Inc., CT, United States; ^2^Boehringer Ingelheim International GmbH, Ingelheim am Rhein, Germany

## Abstract

**Introduction:**

The 10-item Montgomery–Åsberg Depression *Rating* Scale (*MADRS*) measures different dimensions of depression symptomatology. Digital traits may generate deeper understanding of the MADRS subscales and provide insights about depression symptomatology.

**Objectives:**

To identify digital traits that predict specific MADRS subscales and ascertain which digital traits are important for which MADRS subscales.

**Methods:**

During a Phase II decentralised clinical trial in major depressive disorder (MDD), patients completed the MADRS and used AiCure (LLC, New York, NY, USA), a smartphone application, to complete image description tasks at baseline. Digital measurements identified from the literature as relevant to MDD symptomatology were conducted using audio and video data derived from the image description tasks. Digital measurements included speech (rate, sentiment and first-person singular pronouns), vocal acoustics (intensity, pause fraction and fundamental frequency), facial expressivity (regional facial movement) and head pose (Euclidean and angular head movement). Digital traits analysis involved data pre-processing followed by machine learning (ML) using Elastic Net, Decision Tree, and Random Forest models; model performance was evaluated using 5-fold cross-validation and mean absolute error (MAE). Important digital traits were calculated by percentage change in MAE after permuting a specific variable. Important digital traits for the MADRS Apparent Sadness subscale score were mapped to defined, interpretable domains.

**Results:**

The ML model predictions varied for different MADRS subscales (**Table**). Overall, Elastic Net and Random Forest models outperformed Decision Tree across all subscales scores other than suicidal thoughts. Half of the literature-based digital traits contributed to the prediction of ≥1 MADRS sadness sub-scale score. The important digital traits for the Apparent Sadness subscale score could be mapped to 4 domains (**Figure**); this aligned with findings from the literature.

**Image:**

**Figure:**
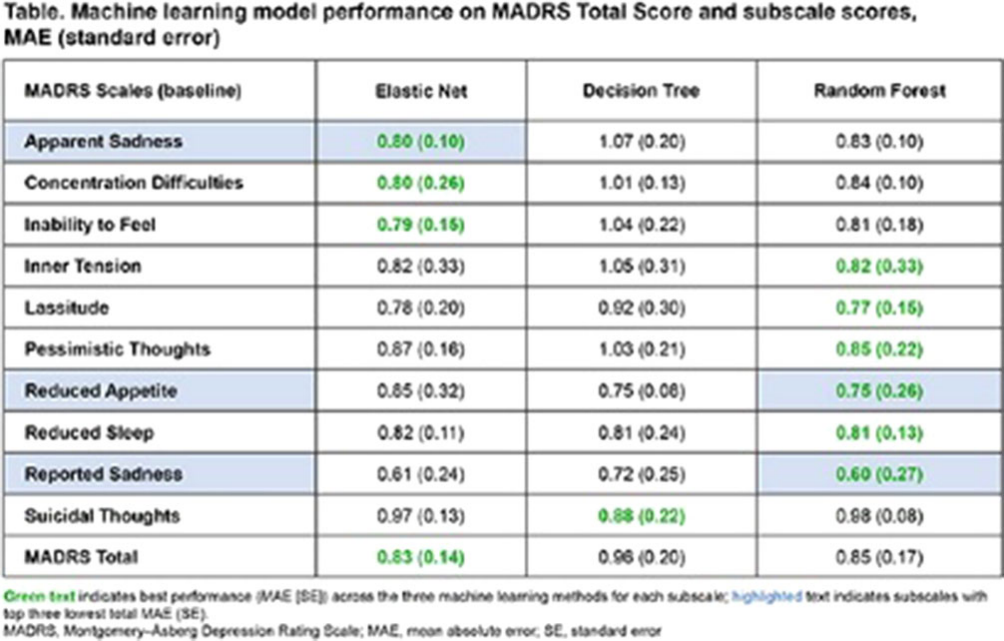

**Image 2:**

**Figure:**
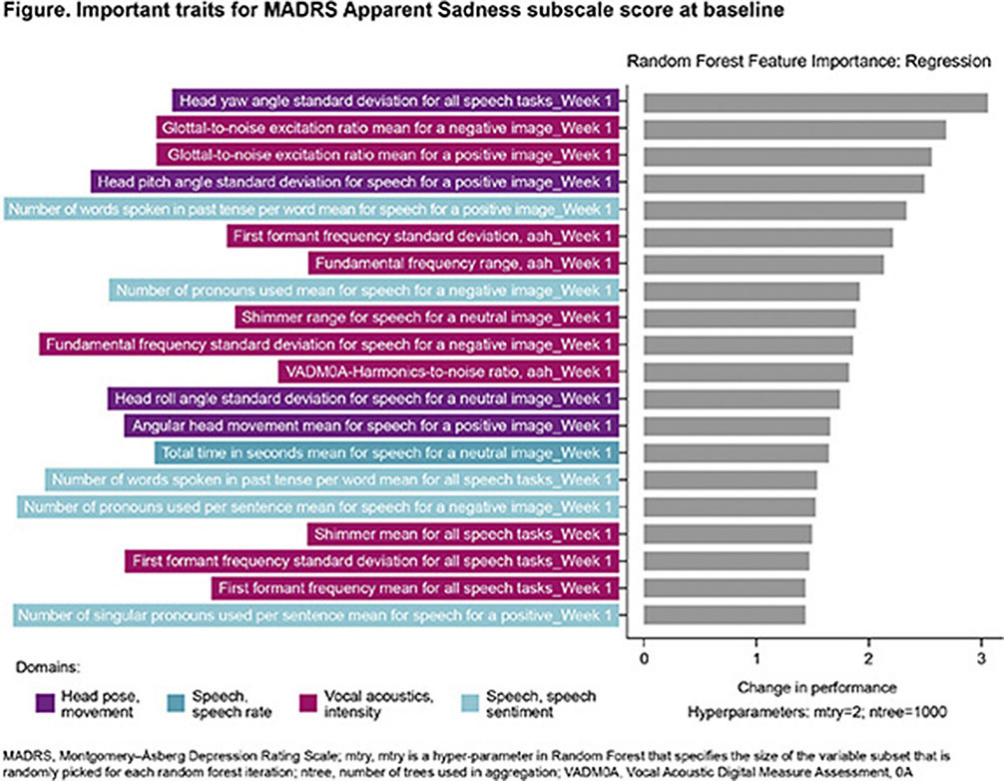

**Conclusions:**

Digital traits collected from patients with MDD were able to predict certain MADRS subscales better than others.

**Funding:**

Boehringer Ingelheim.

**Disclosure of Interest:**

Z. Zhu Employee of: Boehringer Ingelheim Pharmaceuticals, Inc., Y. Wu Employee of: Boehringer Ingelheim Pharmaceuticals, Inc., J. Seidel Employee of: Boehringer Ingelheim International GmbH, D. Roy Employee of: Boehringer Ingelheim Pharmaceuticals, Inc., E. Salzmann Employee of: Boehringer Ingelheim International GmbH

